# Curcumol Synergizes with Cisplatin in Osteosarcoma by Inhibiting M2-like Polarization of Tumor-Associated Macrophages

**DOI:** 10.3390/molecules27144345

**Published:** 2022-07-06

**Authors:** Jincheng Wang, Jialu Jin, Ting Chen, Qian Zhou

**Affiliations:** 1 Center for Drug Safety Evaluation and Research, Zhejiang Province Key Laboratory of Anti-Cancer Drug Research, College of Pharmaceutical Sciences, Zhejiang University, Hangzhou 310058, China; wangjincheng@zju.edu.cn (J.W.); 0921544@zju.edu.cn (J.J.); 2 Department of Pharmacy, Hangzhou Medical College, Hangzhou 310053, China; 2009000003@hmc.edu.cn

**Keywords:** osteosarcoma, CDDP, curcumol, chemoresistance, M2-like macrophages

## Abstract

Osteosarcoma is the most prevalent bone cancer, and chemotherapy is still an indispensable treatment in its clinical practice. Cisplatin (CDDP) has become the most commonly used agent for osteosarcoma, although the outcomes of CDDP chemotherapy remain unsatisfactory because of frequent resistance. Here, we report on a promising combination therapy where curcumol, a bioactive sesquiterpenoid, enhanced CDDP-induced apoptosis to eradicate osteosarcoma cells, and revealed that M2-like macrophages might be the underlying associated mechanisms. First, we observed that curcumol enhanced the CDDP-mediated inhibition of cell proliferation and augmented the apoptosis in osteosarcoma cell lines. Curcumol contributed to preventing the migration of osteosarcoma cells when combined with CDDP. Moreover, this drug combination showed more potent tumor-growth suppression in the orthotopic transplantation of osteosarcoma K7M2 WT cells. We then estimated chemotherapy-associated drug-resistant genes, including ABCB1, ABCC1 and ABCG2, and found that curcumol significantly reversed the mRNA levels of CDDP-induced ABCB1, ABCC1 and ABCG2 genes in the tumor tissue. Moreover, M2-like macrophages were enriched in osteosarcoma tissues, and were largely decreased after curcumol and CDDP treatment. Taken together, these findings suggest that curcumol inhibits the polarization of M2-like macrophages and could be a promising combination strategy to synergize with CDDP in the osteosarcoma.

## 1. Introduction

Osteosarcoma (OS) is the most common primary malignant bone tumor, and represents the second leading cause of cancer-related death in children and adolescents [1,2]. Surgery combined with multimodal chemotherapy, comprising doxorubicin, methotrexate and cisplatin (CDDP), is the standard treatment for OS patients [3,4]. CDDP, an alkylating agent, functions in a cell-cycle-independent manner to cause DNA damage and cell death through alkylated DNA adducts [5]. CDDP is a commonly utilized medication in the clinic as the backbone of neoadjuvant and adjuvant chemotherapy. Drug resistance to CDDP, on the other hand, appears to be a severe issue in the treatment of OS [6,7]. Furthermore, the high invasiveness and metastasis of OS is the fundamental reason for its aggressiveness, leading to a significant relapse rate. Patients with OS metastasis, particularly those with lung metastasis, have a five-year survival rate of less than 30% [8,9,10]. Consequently, focusing on the novel potent chemotherapy regimens is constantly a hot topic in the field of osteosarcoma therapy and plays a key role. Better chemotherapies and elucidation of molecular underpinnings of CDDP resistance are especially important, because they could drastically improve CDDP efficacy for osteosarcoma patients.

Many investigations have found that tumor-associated macrophages (TAMs) play a role in cancer chemoresistance [11,12,13]. TAMs can drive tumor angiogenesis, metastasis, and therapeutic resistance as significant components in tumor microenvironments [14,15,16]. TAMs are heterogeneous cells with two polarized phenotypes: traditionally activated (M1 macrophages) and alternatively polarized (M2 macrophages) phenotypes [17]. TAMs in most solid tumors are largely M2 phenotype macrophages, and M2-type TAMs have been associated with poor prognosis in a variety of malignancies, including osteosarcoma [18,19,20,21]. TAMs have been shown to alter cancer-cell therapeutic responses, making M2-TAMs a novel osteosarcoma treatment option.

Natural products are key sources of anticancer medication development due to their chemical variety and low toxicity [22]. Curcumol is a bioactive sesquiterpenoid that has been isolated from a variety of Zingiberaceae plants [23], and it has attracted considerable interest due to its favorable pharmacological activities and few negative effects. Curcumol has also been demonstrated to have antibacterial, antioxidant, anti-inflammatory, and anti-liver fibrosis properties in previous studies [24,25]. Recently, many studies have indicated that curcumol also inhibits cancer-cell proliferation and death in a variety of cancer cells, including breast cancer, lung cancer, gastric cancer, and cervical cancer [26,27,28]. The structure–activity connection of curcumol is influenced by the presence of substituents at positions 8 and 14, and the free hydroxyl and cyclic structure in curcumol boosts its anticancer action [26]. Importantly, curcumol can overcome the resistance to many agents such as cisplatin, TRAIL, and temozolomide in gastric, lung, and brain tumors [27,28,29]. As far as we know, no studies linking curcumol with osteosarcoma have been published. In this study, we demonstrated the potential anticancer activity of curcumol when combined with CDDP in osteosarcoma. Thus, we move forward to investigate the combination effect of curcumol and CDDP on treating osteosarcoma, and further explore the intrinsic molecular mechanism.

In this study, we found that a bioactive sesquiterpenoid, curcumol, exhibited a strong synergistic antitumor effect on osteosarcoma cells when combined with CDDP by using cell proliferation, apoptosis, invasion, and migration assays. Notably, this combination treatment also displays obvious synergism in an orthotopic transplantation model of osteosarcoma K7M2 WT cells. This combination therapy’s improved tumor-growth suppression was due to a reduction in M2-type macrophages. Overall, the ability of curcumol to enhance the sensitivity of CDDP against osteosarcoma was investigated in our study, indicating that this combination therapy could be a promising treatment option for osteosarcoma patients.

## 2. Materials and Methods

### 2.1. Chemicals

Curcumol (purity 99.9%) was purchased from the National Institutes for Food and Drug Control (Beijing, China). Cisplatin was purchased from Hansoh Pharma (Lianyungang, China).

### 2.2. Cell Lines and Cell Culture

The osteosarcoma cell lines K7M2 WT (wild-type), U2OS, MG63, and KHOS cells were purchased from the Cell Bank of Type Culture Collection of the Chinese Academy of Science (Shanghai, China). Cells were grown and frozen as a seed stock as they were in good condition, and cells were cryopreserved in culture medium containing 10% dimethyl sulfoxide (Sigma-Aldrich, St. Louis, MO, USA). All cell lines were passaged every two or three days, and were passaged for a maximum of two months, after which new seed stocks were thawed. Short Tandem Repeat (STR) profiling was used to assess and authenticate all cell lines. K7M2 WT, MG63, and KHOS cells were cultured in DMEM (Gibco, Grand Island, USA), and U2OS cells were cultured in RPMI-1640 (HyClone, Logan, UT, USA) medium supplemented with 10% fetal bovine serum (FBS, Gibco, Grand Island, NE, USA) in a humidified atmosphere of 5% CO_2_ at 37 °C. 

### 2.3. Cell Proliferation Assay

The sulforhodamine B (SRB, Sigma-Aldrich) assay was used to determine cell growth. Briefly, cells were grown in 96-well plates and treated with curcumol at different concentrations, with or without cisplatin (CDDP). After 72 h, the cells were fixed with 10% trichloroacetic acid and stained with 4 mg/mL SRB for 15 min before being washed three times with 1% acetic acid to remove the excess dye. A Multiskan Spectrum (Thermo Electron Co, Vantaa, Finland) was used to measure the optical density of the protein-bound dye at 510 nm, which was dissolved in a 10 mM Tris-base solution. For each well, the calculation formula of the cell survival rate was (A510 treated/A510 control) × 100%.

### 2.4. Nuclear Morphology Analyses

After washing in PBS, the cells were incubated with PBS containing 0.1% Triton and 0.1% 4′, 6-diamidino-2-phenylindole (DAPI, Sigma-Aldrich). A fluorescence microscope was utilized to examine the morphology of the nuclei.

### 2.5. Flow Cytometry

Apoptosis was assessed using a sub-G1 analysis after PI staining. Samples were fixed with 70% ethanol for 30 min at 4 °C, and then resuspended and stained in 10 μg/mL PI and 40 μg/mL RNase A for an additional 30 min. FACSCalibur cytometer (Becton Dickinson, San Jose, CA, USA) apparatus was employed to perform flow cytometric analysis. On the other hand, samples were incubated with anti-FITC-F4/80 and anti-PE-CD209 antibodies (eBioscience, San Diego, CA, USA) according to the manufacturer’s instructions for the F4/80^+^CD209^+^ analysis. At least 1 × 10^4^ cells were analyzed for each sample.

### 2.6. Western Blotting (WB) Analyses

WB analyses were carried out as previously described [30]. Cleaved PARP and cleaved caspase-3 antibodies were purchased from Cell Signaling Technology (Danvers, MA, USA). The GAPDH antibody was purchased from Santa Cruz Biotechnology and used as an internal control. 

### 2.7. Matrigel Invasion Assay

Membranes coated with a Matrigel matrix (BD Science, San Jose, CA, USA) were used in the invasion assay. The cell suspension (2 × 10^4^ cells/mL) was incubated in the top chamber with 0.2 mL DMEM medium containing 10% FBS for 24 h at 37 °C. Invaded cells were then fixed with 100% methanol and stained with crystal violet. Subsequently, the stained cells were photographed and quantified.

### 2.8. Wound-Healing Assay

K7M2 WT cells were seeded in 24-well plates and grown until they reached 70% to 80% confluence. A straight scratch was made with a pipette tip, and an artificial wound was created. Wound field images were acquired. After curcumol or CDDP treatment for 24 h, a phase-contrast microscope (Leica DMI4000B; Leica Microsystems, Buffalo Grove, IL, USA) was used to document wound closure. Adobe Photoshop CS5 was used to conduct the image analysis. Image analysis was performed with Adobe Photoshop CS5 software.

### 2.9. Orthotopic Transplantation

All animal studies were carried out on female BALB/c mice aged 4 to 5 weeks (National Rodent Laboratory Animal Resource, Shanghai, China). The animal research was authorized by the Animal Research Committee at Hangzhou Medical College, and animal care was provided in accordance with institutional procedures. For tumor formation, K7M2 WT cells (1 × 10^5^) were injected intraosseously into the proximal tibia and distal femur [18]. Mice were divided into 4 groups: untreated control, curcumol (30 mg/kg, oral, daily) alone, CDDP (6 mg/kg, i.p., once per week) alone, and curcumol (30 mg/kg, oral, daily) + CDDP (6 mg/kg, i.p., once per week). 

### 2.10. Immunofluorescence

The tumor samples were subjected to immunofluorescence examination, as previously described [31]. Briefly, cryosections were fixed and permeabilized. Primary antibodies against F4/80 and CD209 (purchased from BD Biosciences, San Jose, CA, USA) were employed, followed by labeling with secondary antibodies conjugated with Alexa Flour 488 or 594 (purchased from BD Biosciences). DAPI staining was used to visualize the nuclei. 

### 2.11. Preparation of BMDM

Isolation of bone marrow was carried out as previously described [14]. Additionally, bone marrow-derived macrophages (BMDMs) were differentiated from bone marrow cells by adding M-CSF (Cell Signaling Technology, Beverly, MA, USA). BMDMs were rinsed with DMEM to eliminate nonadherent cells after 3 days of incubation, and then incubated with 10 ng/mL IL-13 for an additional 5 days.

### 2.12. qPCR

Quantitative real-time PCR analysis was performed using TAKARA SYBR Premix EXTaq^TM^. Reaction mixtures containing SYBR Green were prepared following the manufacturer’s protocol. The primer sequences used for the quantitative RT-PCR were as follows: MRC-1, forward: 5′-AGGGACCTGGATGGATGACA-3′; reverse: 5′-TGTACCGCACCCTCCATCTA-3′; PPAR-γ, forward: 5‘-TTCGATCCGTAGAAGCCGTG-3′; reverse: 5‘-TTGGCCCTCTGATGAGGA-3′; ABCB1, forward: 5′-TGCTGGTTGCTGCTTACA-3′; reverse: 5′-GCCTATCTCCTGTCGCATTATAG-3′; ABCC1, forward: 5′-GGTACCTGTGCTGGTGAATAA-3′; reverse: 5′-TAGGCTTGCTGGGATCTTTG-3′; ABCG2, forward: 5′-GATGAACTCCAGAGCCGTTAG-3′; reverse: 5′-CGGACTAGAAACCCACTCTTTAC-3′; ACTIN, forward: 5‘-GGTCATCACTATTGGCAACG-3′; reverse: 5′-ACGGATGTCAACGTCACACT-3′. β-Actin was used as an internal control.

### 2.13. GSE Datasets

We used GSE12865 and GSE14395 from the GEO public resource (http://www.ncbi.nlm.nih.gov/geo/, GSE12865 Public on 8 September 2009, GSE14395 Public on 11 September 2009) to analyze M2 macrophage marker genes. The selection of GSE datasets was in accordance with PRISMA guidelines [32]. A PRISMA flowchart is shown in Figure S1 (Supplementary Materials).

### 2.14. Statistical Analysis

For all the parameters measured, all the samples’ values in different experimental conditions were averaged, and the standard deviation (SD) was calculated. ANOVA or Student’s unpaired two-tailed t-test were used to examine the statistical significance of differences between groups. * indicates that the results significantly differed from the control (* *p* < 0.05, ** *p* < 0.01, *** *p* < 0.001).

## 3. Results

### 3.1. Curcumol Sensitizes Osteosarcoma Cell Lines to CDDP-Induced Cell Proliferation Inhibition

**Figure 1:**
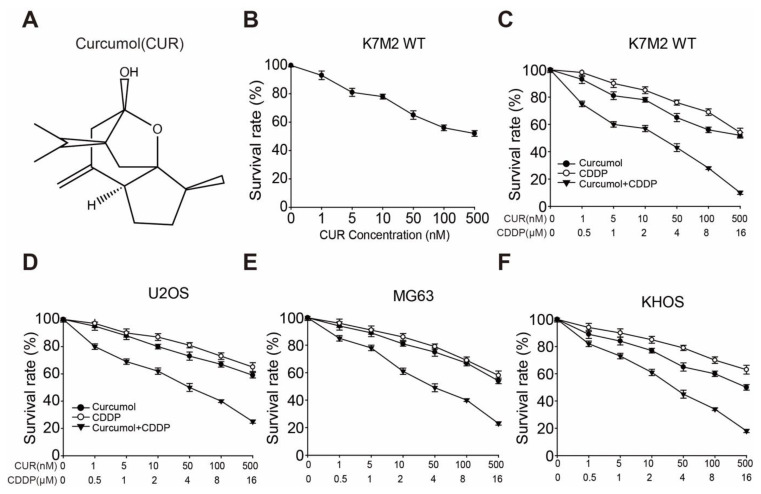
Curcumol increased CDDP-induced cell proliferation in osteosarcoma cells. (**A**) The structural formula of curcumol. (**B**) K7M2 WT cell proliferation was determined using the SRB assay; serial concentrations of curcumol were used to treat cells for 3 days. (**C**–**F**) Cell-proliferation inhibitory activities in osteosarcoma K7M2 WT (**C**), U2OS (**D**), MG63 (**E**), and KHOS (**F**) cells were investigated through SRB assays. Cells were seeded in 96-well plates and treated with serial concentrations of curcumol, CDDP, or combinations of both for 72 h. Dose–response curves for curcumol, CDDP, or combination treatment are displayed. Each experimental value is a result of triplicate repeats, and the error bars indicate the standard deviation (SD).

The structural formula of curcumol is shown in Figure 1A. To determine the optimal concentration of curcumol required to inhibit cell growth in osteosarcoma cells, we determined serial concentrations of curcumol in K7M2 WT cells using the sulforhodamine B (SRB) assay. As shown in Figure 1B, curcumol (0–500 nM) inhibited K7M2 WT cell proliferation in a dose-dependent manner at 72 h. Next, to assess whether curcumol and CDDP exhibited synergistic inhibition effects on the cell proliferation of osteosarcoma, we set a series of concentrations of either drug for 24, 48, and 72 h, and revealed that curcumol or CDDP exerted weak proliferation inhibition at the 24 h point (Figure S2A, Supplementary Materials), whereas curcumol exhibited obvious synergy with CDDP in K7M2 WT cells for 48 h (Figure S2B, Supplementary Materials) and 72 h (Figure 1C). After 72 h, the IC50 values of curcumol alone and CDDP alone were greater than 500 nM and 16 μM, respectively. Upon combination treatment, the IC50 values of curcumol and CDDP were decreased to 14.85 nM and 2.22 μM, respectively (Figure S2C, Supplementary Materials). The proliferation inhibition effect of curcumol combined with CDDP group was similar compared with CDDP + 1 μM ADR (Adriamycin, used as a positive control) (Figure S2D, Supplementary Materials). Moreover, a K7M2 WT/CDDP-resistant cell line was used to evaluate the proliferation efficacy of curcumol, and the results showed that curcumol could overcome the CDDP resistance (Figure S3, Supplementary Materials). Furthermore, to confirm the combination effect of curcumol with CDDP, we employed three widely used human osteosarcoma cell lines, U2OS, MG63, and KHOS [33,34]. As expected, the cell proliferation of each cell line was synergistically suppressed by the combination therapy (Figure 1D–F). Additionally, the IC50 values of curcumol and CDDP were decreased from >500 nM and >16 μM to 37.67 nM and 3.84 μM in U2OS, 41.41 nM and 4.04 μM in MG63, as well as 28.3 nM and 3.24 μM in KHOS (Figure S2C, Supplementary Materials). These results suggest that curcumol can sensitize the CDDP-induced cell-proliferation inhibition of osteosarcoma.

### 3.2. Curcumol Enhanced CDDP-Induced Cell Apoptosis in Osteosarcoma Cells

**Figure 2:**
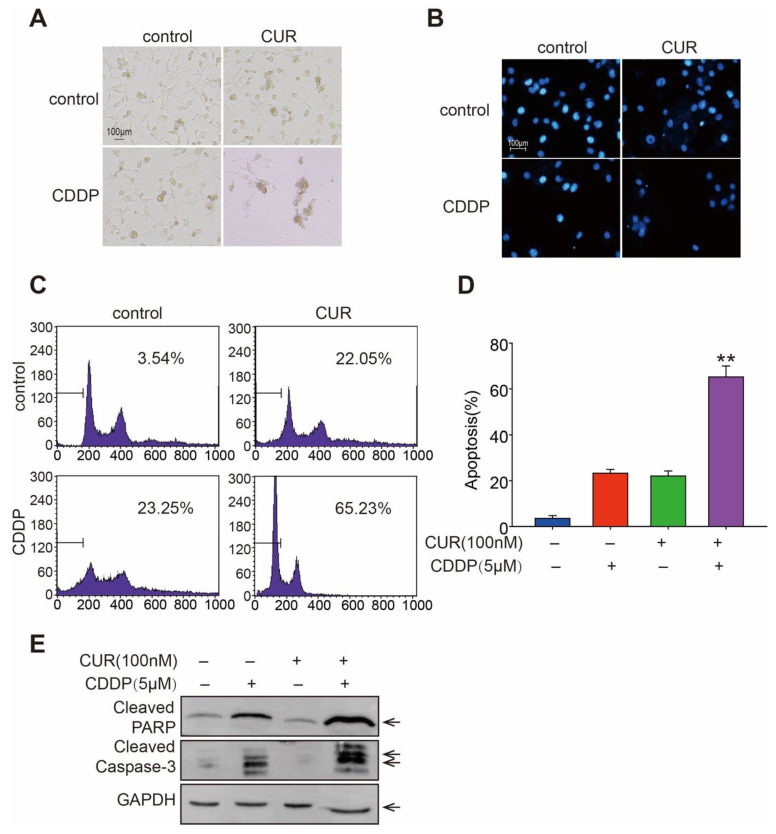
Curcumol enhanced CDDP-induced cell apoptosis in K7M2 WT osteosarcoma cells. (**A**) Cell morphologies were shown in bright-field images after treatment with curcumol, CDDP or a combination of both for 48 h. (**B**) Curcumol, CDDP, or both were employed to treat K7M2 WT cells for 48 h. The cells were then labeled with DAPI, and fluorescence microscopy was used to analyze the nuclear alterations. (**C**) Cells were harvested after being exposed to drugs as described in (**B**), and a PI (propidium iodide) staining experiment was performed and evaluated by flow cytometry. (**D**) Statistical analysis of apoptosis cells in (**C**). ** *p* < 0.01; Student’s *t* test. (**E**) Western blotting for cleaved caspase-3 and cleaved PARP in treated K7M2 WT cells.

The effects of curcumol with CDDP on cell death as well as apoptosis-inducing abilities on K7M2 WT cells were then investigated. When compared with curcumol or CDDP mono-treatment, the combination group showed changes in cell morphology and nuclei, as well as DNA condensation (Figure 2A,B). Moreover, apoptosis induced by curcumol (100 nM) and/or CDDP (5 μM) was determined using flow cytometry analysis after PI labeling, in which the concentration used were similar to the IC50 values at 48 h. As shown in Figure 2C,D, although the monotreatment caused only 22.05% (curcumol) or 23.25% (CDDP) apoptotic K7M2 WT cells, the treatment with curcumol in combination with CDDP induced 65.23% of K7M2 WT cells to experience apoptosis. Activated caspases are essential for promoting the apoptotic cascade. Thus, we looked into the effects of curcumol, CDDP, and their combination on cleaved caspases-3 and cleaved PARP protein levels in K7M2 WT cells. The results showed that curcumol and CDDP combined treatment further enhanced protein levels of cleaved-caspase-3 and cleaved-PARP, compared with mono-treatments (Figure 2E). Our findings indicate that curcumol significantly increases CDDP-induced osteosarcoma cell apoptosis, suggesting that it might be potentially beneficial for treating osteosarcoma in the clinic.

### 3.3. Curcumol and CDDP Combination Treatment Prevents Cell Invasion and Migration

**Figure 3:**
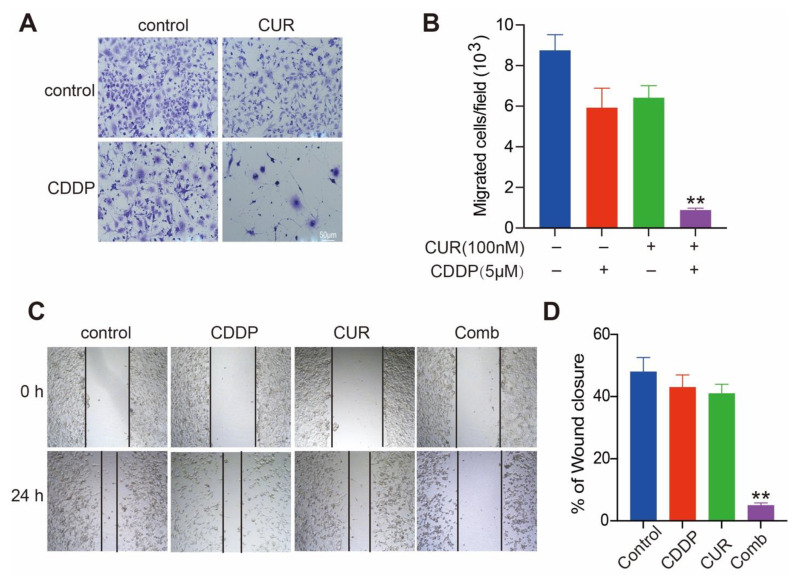
Curcumol combined with CDDP prevents K7M2 WT cells from invasion and migration. (**A**,**B**) Curcumol combined with CDDP suppresses cell invasion. Curcumol was administered to K7M2 WT cells in the absence or presence of CDDP for 24 h, and the cell invasion ability was measured using a Matrigel invasion assay. (**A**), Representative images for Matrigel invasion assay are displayed. (**B**), Quantification of the invaded K7M2 WT cells. Results are presented as the mean ± SD from three independent assays. **, *p* < 0.01, unpaired two-tailed *t* test (vs. untreated control). (**C**,**D**) Curcumol combined with CDDP inhibited wound closure on K7M2 WT cells. Curcumol, CDDP, or both were added to straightly scratched K7M2 WT cells for 24 h, and cell migration across this artificial wound was measured. (**C**), Representative images for wound-healing assay are presented. (**D**), Quantification of the wound area. After image analysis, the gap size at 0 h was set to 100% and the percentage of closed wound after 24 h was calculated. **, *p* < 0.01, unpaired two-tailed *t* test (vs. untreated control).

Next, we investigated the impact of curcumol combined with CDDP on the osteosarcoma metastasis, which is an important cause of death. In the Matrigel invasion assay, noninvasive cells are blocked from migrating through the pores of Transwell plates, while invasive cells destroy the matrix and move through the Matrigel layer. We found that curcumol and CDDP both showed modest inhibitory effect on the invasion of K7M2 WT cells. However, curcumol and CDDP combination significantly reduced the invasion of K7M2 WT cells (Figure 3A,B). Wound-healing assays assess the migration ability and are important methods to assess tumor metastasis; thus, we next evaluated the effect of curcumol and CDDP on the migration of K7M2 WT cells by wound-healing assay. When compared with the control group, curcumol or CDDP monoadministration did not obviously delay the wound closure, whereas the combination of curcumol and CDDP markedly inhibited the wound-closure ability of K7M2 WT cells (Figure 3C,D). Taken together, our results suggest that the combination of curcumol and CDDP might decrease osteosarcoma cell invasion and migration, which further confirms the synergistic antitumor effect of curcumol and CDDP on osteosarcoma.

### 3.4. Curcumol and CDDP Combination Therapy Arrests Tumor Growth in a K7M2 WT Orthotopic Transplantation Model

**Figure 4:**
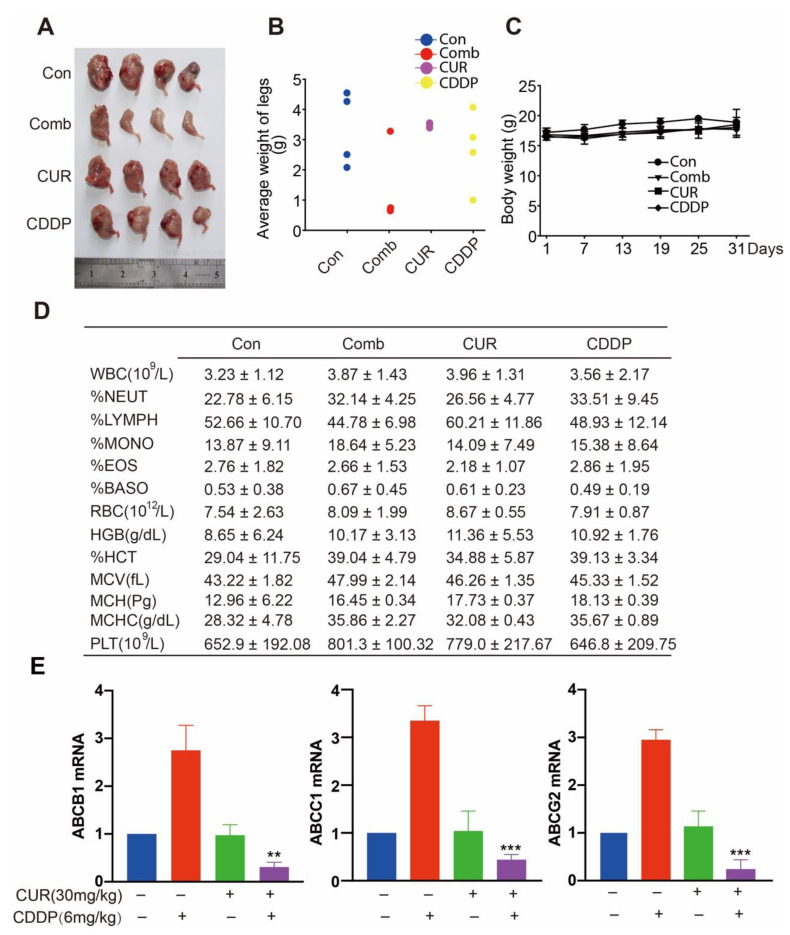
Curcumol synergized with CDDP to inhibit tumor growth in osteosarcoma mouse model. To measure tumor formation, BALB/c mice were intrafemorally injected with 1 × 10^5^ K7M2 WT cells and euthanized after 4 weeks. (**A**) Representative images of osteosarcoma tumor formation. (**B**) The weight of the tumor-bearing legs. (**C**) Mice body weights were monitored weekly, and the results are shown as the mean ± standard deviation (SD). (**D**) Whole-blood test. (**E**) qPCR quantification of drug-resistance genes in osteosarcoma tissues treated with curcumol in the presence or absence of CDDP. **, *p* < 0.01, ***, *p* < 0.001, unpaired two-tailed *t* test (vs. untreated control).

To determine whether curcumol and CDDP synergized in vivo, we evaluated the antitumor activity of curcumol and CDDP in combination in a K7M2 WT orthotopic transplantation mouse model. After the implantation of cells into BALB/c mice, 6 mg/kg CDDP and 30 mg/kg curcumol were administered i.p. twice per week for 31 days. As shown in Figure 4A,B, compared with the control group, curcumol or CDDP administration resulted in weak-to-moderate tumor weight inhibition, whereas simultaneous curcumol and CDDP treatment resulted in significantly slower tumor growth. Moreover, the body weight among these four groups did not obviously alter (Figure 4C). In addition, we also conducted whole-blood assays, and the data showed no significant differences among these four groups (Figure 4D). Finally, in order to evaluate the underlying mechanism of this drug combination, we detected three drug-resistance genes, including ABCB1 (P-glycoprotein, P-gp), ABCC1 (multidrug-resistance protein 1, MRP1) and ABCG2 (mitoxantrone-resistance protein, MXR), which are the most clinically significant ABC transporters that cause multidrug resistance during cancer therapy [35,36]. Figure 4E demonstrated that CDDP markedly increased the mRNA levels of ABCB1, ABCC1, and ABCG2, whereas curcumol combined with CDDP could significantly inhibit these three drug-resistance genes in the osteosarcoma tissues. Thus, we concluded that combined therapy with curcumol plus CDDP produces much more potent antitumor effects than in vivo monotherapies.

### 3.5. M2-like Macrophages Are Decreased in the Combination Treatment of Curcumol and CDDP

**Figure 5:**
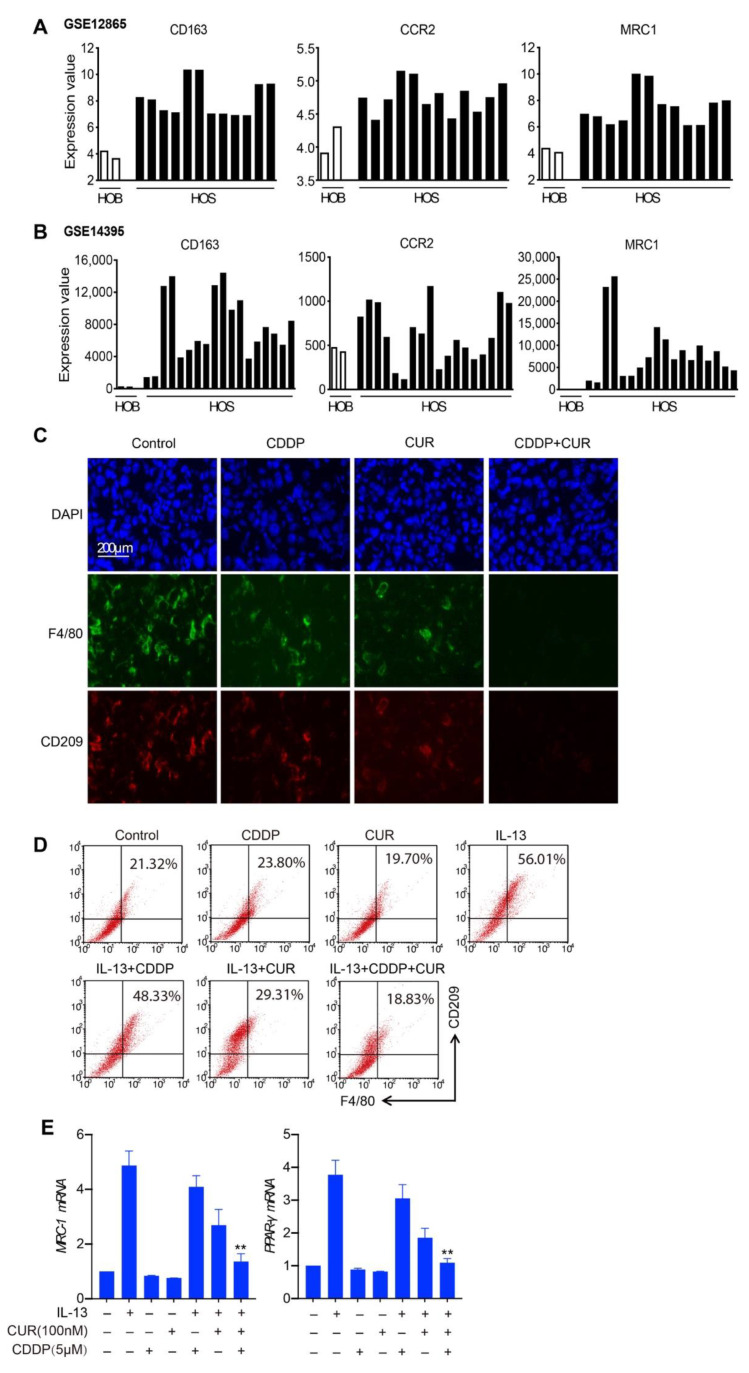
Curcumol combined with CDDP reduced enrichment with M2-like macrophages. (**A**) and (**B**) The expression levels of M2-like macrophages marker genes in normal human osteoblasts and human osteosarcoma tumors from the GSE12865 (**A**) and GSE14359 (**B**) datasets, respectively. HOB: normal human osteoblasts, human sample number = 1 in both GSE12865 and GSE14395, two bars represent two replicates for the sample; HOS: human osteosarcoma tumor, patient sample numbers = 6 in GSE12865 and 9 in GSE14395, every two bars represent two replicates for each sample. Each bar represents the gene-expression value from one patient sample or its replicate. (**C**) Representative images of F4/80^+^ macrophages and CD209^+^ TAMs in the tumor sections analyzed by immunofluorescence. (**D**) FACS analysis of F4/80^+^CD209^+^ cells polarized from BMDM cells treated with IL-13 alone or together with CDDP and/or curcumol. (**E**) qPCR quantification of *MRC1* and *PPAR-γ* mRNA levels in BMDM cells treated with IL-13 alone or together with CDDP and/or curcumol. **, *p* < 0.01, unpaired two-tailed *t*-test (vs. IL13-treated group).

In a previous study, we found that M2-like macrophages played a major role in osteosarcoma initiation and stemness. Cancer stem cells contribute to chemotherapy-resistance; therefore, we further evaluated the role of M2-like macrophages in this combination regimen. Firstly, we referred to two data sets, GSE12865 and GSE14395, and showed that there were high expression levels of four classical M2 macrophages markers, CD163, CCR2, and MRC1, in human osteosarcoma tissue samples. (Figure 5A,B). Next, we determined the expression levels of CD209 (another M2 macrophage marker) in osteosarcoma tissues from K7M2 WT orthotopic transplantation through an immunofluorescence assay. Histological examination showed that compared with mice transplanted with only K7M2 WT cells, mice treated with curcumol and CDDP were weakly enriched with M2-like macrophages (F4/80^+^CD209^+^) (Figure 5C). Then, we investigated the effect of curcumol and CDDP in the polarization of macrophages, and isolated primary bone marrow-derived macrophages (BMDM). Similar results were observed, as shown in Figure 5D; the percentage of F4/80^+^CD209^+^ cells increased from 21.32% in the control group to 56.01% in IL-13-treated cells. Curcumol decreased IL-13 induced F4/80^+^CD209^+^ expression, from 56.01% in the IL-13 group to 29.31% in the IL-13 and curcumol combination group. Moreover, the group of IL-13, combined with CDDP and curcumol, markedly decreased M2-polarized macrophages (from 56.01% to 18.83%). *MRC-1* and *PPAR-γ* (M2 markers) induced by IL-13 were blocked by CDDP and curcumol combination treatment in BMDM cells (Figure 5E). These results demonstrate that M2-polarized macrophages might contribute to CDDP resistance and that curcumol could synergize with CDDP to reduce M2 macrophage-promoted osteosarcoma resistance.

## 4. Discussion

In the clinic, cisplatin-based treatment is often utilized as a chemotherapeutic criterion in osteosarcoma. However, several combination treatments tested in clinical trials over the last 40 years have failed to enhance the long-term survival rate of osteosarcoma patients [37,38]. After chemotherapy, there appeared to be a bottleneck in the treatment of osteosarcoma, which needed to be addressed as soon as possible [39]. As a result, we recommended a strong combination therapy to eradicate osteosarcoma, as demonstrated in this study. Curcumol could initially, highly sensitize osteosarcoma cell lines to CDDP-induced cell-growth suppression in a time- and dose-dependent manner, especially at 48 h and 72 h time intervals. Furthermore, when CDDP is coupled with curcumol, apoptosis is significantly increased. Curcumol and CDDP, however, clearly slowed osteosarcoma cell movement indicated by transwell and wound-healing assays, which may represent an effective way to prevent the metastasis of osteosarcoma. Furthermore, in an orthotopic transplantation model of osteosarcoma K7M2 WT cells, the combined therapy of curcumol with CDDP significantly inhibited osteosarcoma tumor growth. Then, we determined what our combination therapy’s underlying mechanism was. TAMs were critical for curcumol and CDDP synergism, according to our findings. Curcumol coupled with CDDP inhibited M2-polarized macrophages and reduced M2-type macrophage recruitment in vivo. The inhibition of M2-TAMs has substantial synergistic action when coupled with CDDP, according to our findings. This research could pave the way for new strategies to use CDDP and curcumol in combination as a promising treatment option for osteosarcoma patients.

Curcumol, a pure monomer isolated from Rbizoma Curcumae, has been shown to exhibit anticancer properties in a variety of cancer cells [26,27]. Curcumol has recently been found to improve the sensitivity of chemical medicines such as Adriamycin and 5-FU [40]. Curcumol was demonstrated to have a potent anticancer impact in colon cancer cells by inducing apoptosis, inhibiting proliferation and migration, and improving the therapeutic efficacy of 5-FU. Curcumol also inhibits the phosphatidylinositol 3-kinase (PI3K)/protein kinase B (AKT) pathway, which may increase the sensitivity of gastric cancer cells to cisplatin-based chemotherapies [27]. Curcumol may also alter the development of prostate cancer via regulating the PDK1/AKT/mTOR signaling pathway via miR-9 [41]. Curcumol inhibits the production of programmed cell death-ligand 1 in hepatocellular carcinoma cells via the hypoxia-inducible factor-1 alpha and the signal transducer and activator of transcription 3 signaling pathways, restoring the tumor-killing capabilities of cytotoxic T lymphocytes [42]. These findings imply that curcumol exerts anticancer properties through a variety of mechanisms.

TAMs, as a source of essential components in inflammatory microenvironments, are increasingly being implicated in the occurrence, development, and therapy of viral cancers [43,44,45,46]. After infiltrating tumors, TAMs secrete inflammatory cytokines such as CXCL8, IL-6, and IL-1, which contribute to persistent inflammation [47,48,49]. M2 polarized macrophages provide chemotherapy resistance in cancer cells such as gastric cancer cells, according to the contemporary literature [50,51,52]. Patients with advanced GC (gastric cancer) who acquire resistance to cisplatin currently have few treatment choices in the clinic [53]. Peiming et al. reported that M2-polarized macrophages promote CDDP resistance in gastric cancer cells by the exosomal transfer of functional miR-21 [50]. These data imply that TAM-derived exosomes are an important facilitator of TAM-gastric cancer cell reciprocity, and that targeting exosomal miR-21 from TAM could be a promising adjuvant therapy option for CDDP-resistant GC patients. Curcumol adds to CDDP sensitivity in osteosarcoma cells by inhibiting M2 polarized macrophages, according to this study. However, more research on the mechanism by which curcumol modulates macrophage polarization is needed in the future. Moreover, it is unclear whether microRNA produced by exosomes plays a role in curcumol antagonizing osteosarcoma, and more research is needed.

In a recent study, we found that M2-like macrophages enhance the number of CD117^+^Stro-1^+^ CSCs (cancer stem cells), as well as the expression of potential CSC markers, and promote osteosarcoma cell-sphere formation [18]. Accumulating populations of CSCs have been shown to increase treatment resistance in tumor patients [54,55,56]. TAM-CSC appears to play a role in osteosarcoma progression and chemotherapy response in the current investigation. TAMs play a direct role in tumor initiation and progression via viral mechanisms [57,58]. Curcumol has been shown to reduce M2-skewed macrophages and may also impair the TAM–CSC relationship, allowing chemical medicines to respond more effectively. Further research is needed to determine whether curcumol can impair the CSC features of osteosarcoma cells produced by M2-type macrophages. Taken together, we have proposed M2-polarized TAMs as an appropriate therapeutic target for adjuvant chemotherapy in osteosarcoma. 

## 5. Conclusions

In conclusion, we have demonstrated, for the first time, that curcumol enhances the CDDP-induced apoptosis of osteosarcoma cells both in vitro and in vivo. The combination therapy not only exhibited outstanding efficacy in cell lines but was also effective in an orthotopic transplantation model with well pronounced tumor inhibition ability. These findings supported curcumol’s combination efficacy with other anticancer agents and revealed a possible approach for overcoming chemotherapy resistance. Furthermore, CDDP combined with curcumol suppressed the activation of M2-like macrophages, thereby potentiating the anticancer effects of both CDDP and curcumol, although these molecular mechanisms need to be further studied. Collectively, the combination of CDDP and curcumol improved the observed superior pharmacological effects and shed novel light on the strategy of inhibiting M2 macrophages, indicating new approaches for the clinical chemotherapy regimens in osteosarcoma treatment.

## Data Availability

All data used to support the findings of this study are included within the article and the Supplementary Materials.

## Supplementary Materials

The following are available online at www.mdpi.com/article/10.3390/molecules27144345/s1, Figure S1: Flowchart of inclusion criteria and exclusion criteria for GSE datasets selection according to the PRISMA guidelines, Figure S2. Curcumol synergized CDDP to inhibit cell proliferation in K7M2 WT cells, Figure S3. Curcumol synergized CDDP to inhibit cell proliferation in K7M2 WT/CDDP-resistant cells.
