# Supplementary material for: Curcumol Synergizes with Cisplatin in Osteosarcoma by Inhibiting M2-like Polarization of Tumor-Associated Macrophages
Source: Molecules. 2022 Jul 6;27(14):4345. doi: 10.3390/molecules27144345 (PMC9318016; doi:10.3390/molecules27144345)
Supplement: Supplementary file 1 [file molecules-27-04345-s001.zip › molecules-1704483-supplementary.pdf]

Supplementary Materials

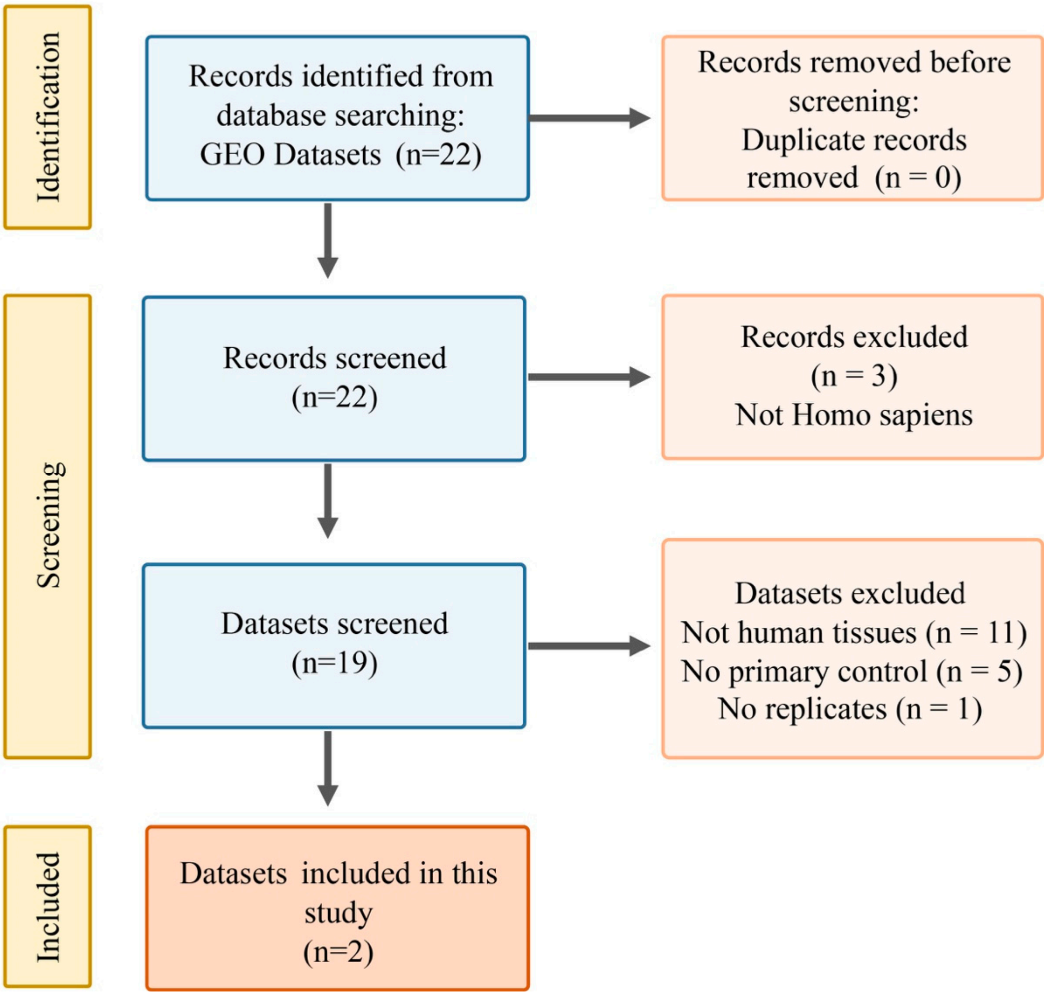

Figure S1. Flowchart of inclusion criteria and exclusion criteria for GSE datasets selection according to the PRISMA guidelines.

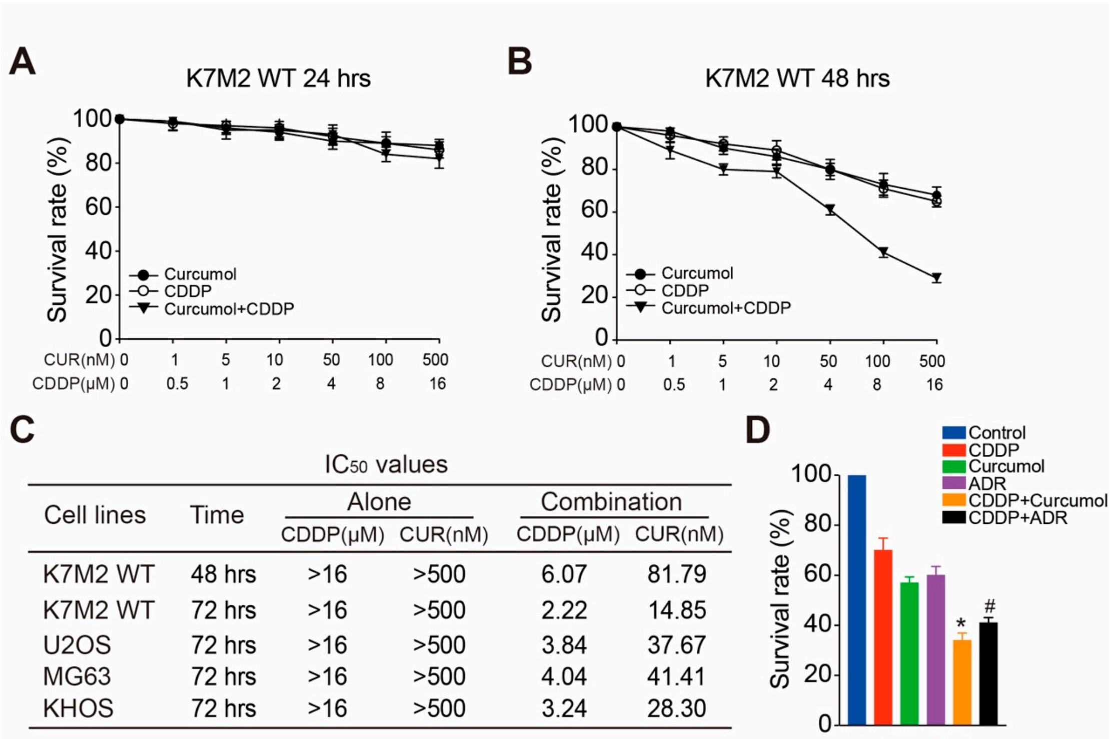

Cell lines	Time	Alone		Combination	
		CDDP(μM)	CUR(nM)	CDDP(μM)	CUR(nM)
K7M2 WT	48 hrs	>16	>500	6.07	81.79
K7M2 WT	72 hrs	>16	>500	2.22	14.85
U2OS	72 hrs	>16	>500	3.84	37.67
MG63	72 hrs	>16	>500	4.04	41.41
KHOS	72 hrs	>16	>500	3.24	28.30

Figure S2. Curcumol synergized CDDP to inhibit cell proliferation in K7M2 WT cells. (A-B) Cell-proliferation inhibitory activities in K7M2 WT cells were investigated by SRB assays. Cells were seeded in 96-well plates and treated with serial concentrations of curcumol, CDDP or combinations of both for 24 h (A) or 48 h (B). Dose-response curves for curcumol, CDDP or combination treatment are displayed. Each experimental value is a result of triplicate repeats, and the error bars indicate the standard deviation (SD). (C) The IC50 values were calculated for the curcumol and CDDP concentrations used in the SRB assays. (D) K7M2 WT cells were seeded in 96-well plates and the groups were set as: control, CDDP (5μM), Curcumol (100nM), ADR (1μM), CDDP (5μM)+Curcumol (100nM), and CDDP (5μM)+ADR (1μM). After 72 h treatment, cells were collected for SRB assay. *$p<0.05$ (CDDP group compared with the CDDP+Curcumol group), # $p<0.05$ (CDDP group compared with the CDDP+ADR group); Student's t test.

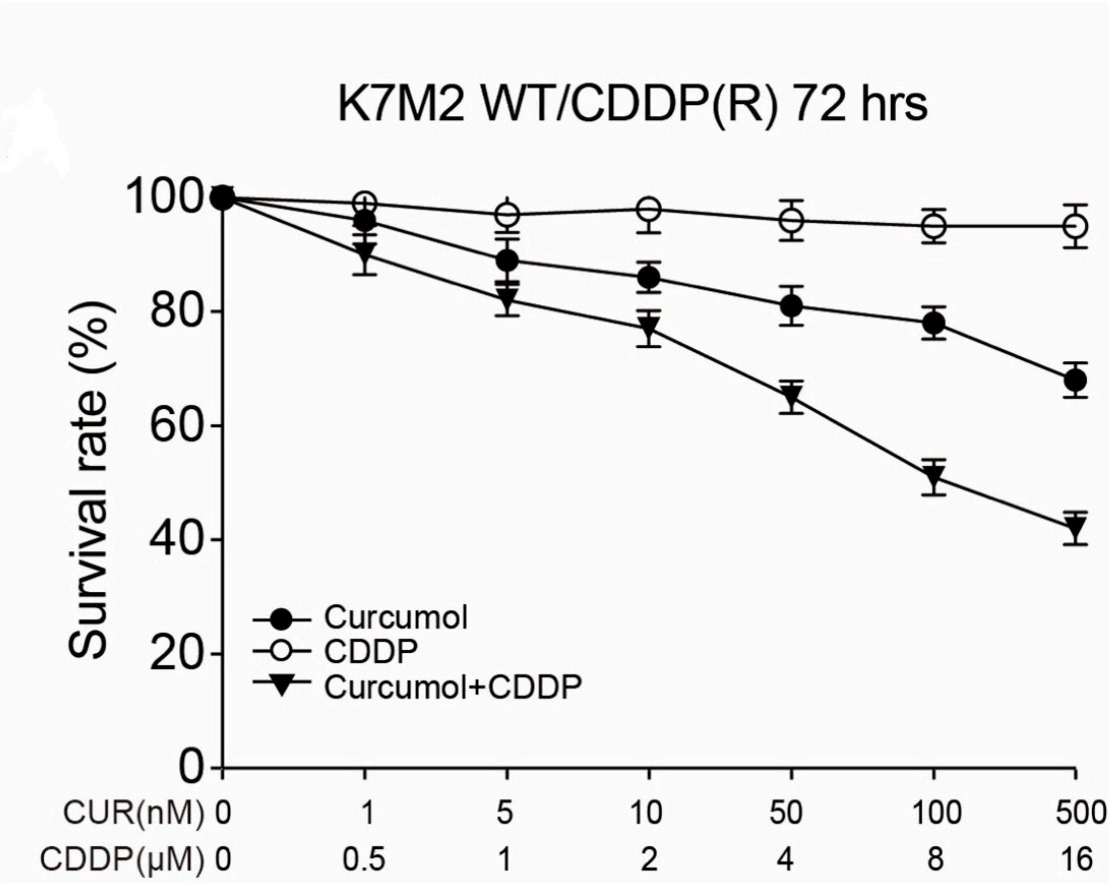

Figure S3. Curcumol synergized CDDP to inhibit cell proliferation in K7M2 WT/CDDP-resistant cells. The drug-resistant cell line (K7M2 WT/CDDP (R)) was obtained by plating K7M2 WT cells onto a 6-well plate and treating with 5μM CDDP for 48h, and then the dead cells were removed with DMEM (Gibco). This step was repeated until the subsequent addition of CDDP did not lead to any further cell death. Finally, the cells obtained were the drug-resistant cell line (K7M2 WT/CDDP-resistant cells). (A) Cell-proliferation inhibitory activities in K7M2 WT/CDDP(R) cells were investigated by SRB assays. Cells were seeded in 96-well plates and treated with serial concentrations of curcumol, CDDP or combinations of both for 72h. Dose-response curves for curcumol, CDDP or combination treatment are displayed. Each experimental value is a result of triplicate repeats, and the error bars indicate the standard deviation (SD).
